# Rollout of rapid point of care tests for antenatal syphilis screening in Ghana: healthcare provider perspectives and experiences

**DOI:** 10.1186/s12913-018-2935-y

**Published:** 2018-02-20

**Authors:** Edward Tieru Dassah, Yaw Adu-Sarkodie, Philippe Mayaud

**Affiliations:** 1 School of Public Health, Kwame Nkrumah University of Science and Technology, Kumasi, Ghana; 2 Department of Obstetrics and Gynaecology, Komfo Anokye Teaching Hospital, Kumasi, Ghana; 3 Department of Clinical Microbiology, School of Medical Sciences, Kwame Nkrumah University of Science and Technology/ Komfo Anokye Teaching Hospital, Kumasi, Ghana; 4 London School of Hygiene and Tropical Medicine, London, UK

**Keywords:** Rollout, Health care providers, Point of care tests (POCTs), Antenatal syphilis screening, Ghana

## Abstract

**Background:**

Effective implementation of rapid point of care tests (POCTs) for antenatal syphilis screening especially in settings where antenatal care attendance is high, can significantly increase screening coverage and treatment uptake. The operational challenges of introducing rapid syphilis POCTs at scale needs to be investigated. This study explores healthcare providers’ experiences and challenges in antenatal syphilis screening following the national rollout of rapid syphilis POCTs in Ghana.

**Methods:**

Prior to the main study, we undertook a desk review of key syphilis policy documents, and conducted key stakeholder interviews and a baseline survey of syphilis screening practices. Antenatal syphilis screening had been poorly implemented mainly due to inadequate technical and logistic support, and lack of monitoring and supervision. For the main research, semi-structured interviews were conducted with 51 purposively selected healthcare staff involved in antenatal syphilis screening in 15 health facilities in three regions, representative of all levels of healthcare in Ghana and two regional programme coordinators, at least four months after the rollout. The interviews were supplemented with an audit of the conduct of antenatal care, syphilis-related supplies and other maternal and newborn interventions. Qualitative data were coded and analysed using Nvivo software.

**Results:**

Syphilis screening with rapid POCTs was integrated into antenatal care in almost all (13/15) the facilities surveyed. Testing and treatment were offered free of charge to pregnant women, their partners and babies. In most facilities, midwives were performing syphilis tests together with HIV tests. Operational challenges included: inadequate training and lack of refresher training, lack of clear testing guidelines, clear channels of communication, supervision, and guidance on treatment and referral procedures, frequent stockouts of, or expired test kits, staff overload, and poor documentation of test results and treatment.

**Conclusion:**

Although syphilis screening with rapid syphilis POCTs was integrated into antenatal care, key challenges, particularly around supply chain and supervision, need to be addressed to improve and sustain such a programme.

## Background

Globally, over half a million entirely preventable perinatal deaths are attributable to maternal syphilis each year [1]. These adverse pregnancy outcomes can be averted through effective screening and treatment of women who are positive for syphilis during pregnancy [1]. However, even in countries where antenatal care (ANC) coverage is high and antenatal syphilis screening exists as a national policy, routine screening may not be well implemented at scale, commonly due to the organisation of health services, operational challenges and cost of syphilis testing [2]. Recent studies on the use of rapid syphilis point of care tests (POCTs) for antenatal syphilis screening in clinic settings have demonstrated that these tests can effectively increase screening coverage and treatment uptake [3].

National reproductive health policy promotes syphilis screening as an integral component of ANC in Ghana [4]. However, antenatal syphilis screening had been poorly implemented and pregnant women in many antenatal clinics were not being screened and treated for syphilis [5]. For example, only 4% of health facilities nationwide offered syphilis testing as a routine component of ANC and laboratory tests for syphilis were not widely available [6]. About three quarters of health care providers in the Greater Accra and Central regions did not have basic training on syphilis screening and providers’ knowledge of the management of syphilis in pregnancy was particularly low [7]. Reasons cited for the poor implementation of the policy included; poor dissemination and inadequate guidance for implementation, lack of training, and insufficient logistic support [5, 7].

Consequently, following reported dramatic increases in maternal syphilis seroprevalence during sentinel surveillance, the Ghana Health Service (GHS) rolled-out rapid syphilis POCTs for routine antenatal screening in public healthcare facilities throughout the country from October 2009. The tests and treatment are offered completely free of charge to pregnant women, their partners and babies. Antenatal care attendance is high in Ghana (76% for at least 4 visits, and 96% for at least one visit) [8], suggesting that effective implementation of rapid syphilis POCTs and treatment of seropositive women during pregnancy can prevent the consequences of maternal syphilis.

While rapid POCTs are expected to increase syphilis screening and treatment coverage, the operational challenges of introducing syphilis POCTs at scale have not been investigated. In this study, we present the perspective of healthcare providers in public health facilities in selected regions of Ghana in relation to their experiences and challenges following a national rollout of rapid syphilis POCTs in Ghana. We sought to determine if previous known challenges were addressed, and also identify any new challenges associated with the current rollout at the facility level. The performance of the programme was also compared with three other recommended antenatal interventions, namely prevention of mother-to-child transmission (PMTCT) of HIV, intermittent preventive treatment of malaria in pregnancy (IPTp), and prevention of maternal and neonatal tetanus (tetanus toxoid), as tracer control interventions. The results of the study were meant to inform national policy of challenges in the existing programme that needed to be addressed to improve antenatal syphilis screening coverage with POCTs and treatment of seropositive cases.

## Methods

### Baseline survey and study context

Prior to the main research, a baseline survey of key syphilis policy documents, stakeholders and syphilis screening practices was carried out by one author, ETD. This formative research was conducted to assess the existence of a national policy for, and practise of antenatal syphilis screening in Ghana prior to the national rollout of syphilis POCTs.

Semi-structured interviews were conducted with 15 stakeholders who were purposively selected from nine organizations at the national level including various divisions/programmes of the GHS and Ministry of Health, and some multilateral agencies relevant to sexually transmitted infection (STI) control and policy implementation. Within each organization, the director, manager/coordinator or senior technical advisor was interviewed. A desk review of syphilis policy documents was also undertaken during this phase of the research.

The desk review and key stakeholder interviews indicated that syphilis screening had been a long-standing component of antenatal care in Ghana, and an integral part of the reproductive health policies of the country since 1996. The policy mandates that all pregnant should be screened for syphilis during antenatal care [4, 9]. However, at the time of this baseline survey, implementation had been poor and screening had not been routinely practised in many antenatal clinics due to several challenges. These included under-appreciation of the problem of syphilis, lack of confidence in the current surveillance strategy, confusion over responsibilities for antenatal syphilis control due to poor collaboration between complementary agencies, insufficient logistic and technical support, poor documentation and lack of indicators for monitoring and evaluation.

A rapid audit was undertaken in 10 facilities in three regions, Central, Ashanti and Northern Regions. Only 3 of the 10 facilities routinely screened pregnant women for syphilis. In these facilities, pregnant women were referred to the laboratory within the facility, where blood samples were taken and syphilis testing done using POCTs. The 3 facilities used different kinds of POCT kits, purchased individually from allocated funds. Stockouts of POCTs was rare. Two of the facilities were in the Central region, and routine testing had been introduced prior to the national rollout due to the persistent high prevalence of syphilis during the national HIV/syphilis sentinel survey. Only one tertiary facility had equipment to perform conventional syphilis testing with rapid plasma reagin (RPR) or Venereal Disease Research Laboratory (VDRL) tests, and also regularly stocked benzathine penicillin for syphilis treatment. Syphilis test results were routinely documented in the ANC registers of the 2 facilities in the Central Region. There was no column in the register for recording treatment. None of the facilities had specific syphilis screening and treatment guidelines.

### Selection of regions and health facilities

We conducted a qualitative research aiming to be inclusive of all health regions of Ghana and to be representative of all levels of health service delivery in the country. We therefore designed a multilevel purposive sampling frame. A detailed description of the selection of the regions, districts and health facilities has been given elsewhere [10]. Briefly, the Central, Ashanti and Northern Regions (the same regions that were selected for the baseline survey) were purposively selected to reflect high, moderate and low syphilis seroprevalence regions respectively, since antenatal syphilis screening practices could be influenced by reported syphilis seroprevalence. The reported syphilis seroprevalence were based on the 2004–2009 HIV/syphilis sentinel surveys. Altogether 15 health care facilities were selected across the different levels of health care delivery in the three regions. The study is reported according to the consolidated criteria for reporting qualitative research (COREQ) [11].

### Healthcare provider interviews and audit of the conduct of ANC

Face-to-face semi-structured interviews were conducted in English between four and fifteen months after the syphilis POCT rollout had occurred (from August 2010 to February 2011) with purposively selected healthcare staff involved in antenatal syphilis screening in each of the 15 health facilities and regional STI coordinators, after obtaining written informed consent. The staff included doctors, physician assistants, midwives, pharmacists/dispensing technicians and laboratory scientists. Within each health facility, the head of the facility or Obstetrics and Gynaecology department (for regional and teaching hospitals), unit head for maternity and/or a midwife from the antenatal clinic, a laboratory technician and a pharmacist or dispensing technician were purposively selected for interview. The midwives and laboratory personnel performed antenatal syphilis screening using POCTs. The pharmacists/dispensing technicians were chosen to assess issues relating to availability and stock levels of benzathine penicillin. The facility or unit heads (medical superintendents, physician assistants or midwives) and doctors involved in STI management (medical officers) were selected because of their role in the training, supply and use of syphilis POCTs and treatment of syphilis seropositive cases. All interviews were conducted within the health facilities or offices by one author (ETD), who was a medical practitioner in one of the teaching hospitals, and knew some of the participants. Each interview lasted between 7 and 40 min, depending on the role of the provider in antenatal syphilis screening and treatment. Generally, interviews with the pharmacists/dispensing technologists were the shortest, while those with the midwives and medical officers tended to be much longer. The interviews focused on the training of staff in syphilis screening and management, supply of test kits and drugs, guidance on testing procedures and treatment protocols, documentation of test results and treatment, monitoring and supervision, barriers and challenges to antenatal syphilis screening and ways of improving and sustaining the programme, as well as other recommended national prenatal interventions such as PMTCT of HIV, IPTp and prevention of maternal and neonatal tetanus for comparison as tracer control interventions. The interview guide was developed after a review of the literature, study objectives and research questions and discussion among the research team. All interviews were digitally recorded and supplementary written notes taken as necessary. One staff from each of the selected categories/units in each facility, who was available, was approached for interview and agreed to participate in the study. There was no attempt to stop at theoretical saturation, since we intended to obtain experiences of healthcare providers across the different levels of reported syphilis seroprevalence and healthcare delivery in the country.

In addition, an audit of the conduct of ANC and a comprehensive inventory of testing, treatment and referral guidelines, and stock levels of test kits and treatment supplies for syphilis and the control tracer interventions (including needles and syringes, benzathine penicillin, erythromycin, nevirapine, sulphadoxine pyrimethamine and partner notification cards) was conducted. Two structured observations of the content of health education during ANC, site and timing of syphilis testing (antenatal clinic, onsite laboratory or offsite laboratory), as well as the management of syphilis seropositive cases (including counselling, provision of results and treatment given to clients, their contacts and babies) were carried out in each facility. The availability of syphilis screening and management related guidelines and protocols was also assessed and compared with other recommended antenatal interventions.

### Data management and analyses

Audio recordings were transcribed verbatim on each day of the interview after returning from the field. Transcripts were explored through multiple readings for accuracy and familiarization; a priori themes, key issues, concepts, emerging and recurrent themes were noted at this stage. Predefined themes were developed based on review of the literature, the research objectives and questions and preliminary review of the interview transcripts. The transcripts were then imported into NVivo 8 (QSR International Pty Ltd., Melbourne, Australia), coded and analysed in detail using framework analysis [12]. One script was independently coded line by line by two authors. They then discussed any discrepancies and reached a consensus on the codes to be used for the other transcripts. The rest of the transcripts were then read thoroughly and coded accordingly while paying attention to any new codes that emerged. The codes were then grouped under our predefined themes. The results are presented according to the major themes and sub-themes. Proportions were calculated in percentages for various categories of structured observations, guidelines and stock levels of supplies.

### Trustworthiness

To increase the trustworthiness of the study, we triangulated the information obtained from the various healthcare providers by comparing their experiences across facilities. We also held a peer-debriefing between all authors to discuss the coding process, categories and interpretation of the study data.

### Research ethics

Written informed consent was obtained from all participants including their consent to record the interviews and also quote them. Participation in the study was entirely voluntary and participant anonymity was maintained throughout the processes of interview transcription, data analysis and presentation. The study was approved by the Ghana Health Service Ethical Review Committee and the Committee on Human Research, Publications and Ethics, Kwame Nkrumah University of Science and Technology, Ghana, and the Ethics Committee of the London School of Hygiene and Tropical Medicine, United Kingdom.

## Results

Fifty-one healthcare providers in the 15 healthcare facilities and two regional STI coordinators were interviewed (Table 1); one regional STI coordinator was not available for interview during our visit to that region and could not be contacted afterwards. Antenatal syphilis screening was decentralised to almost all (13/15) the public facilities surveyed. Operational challenges identified included inadequate training, lack of testing guidelines and treatment protocols, frequent stockouts or expired test kits, staff overload, inadequate monitoring and supervision, and issues with documentation of test results and treatment. A summary of the major themes and subthemes, and key findings are summarised in Table 2.

**Table 1:** Regions and level of healthcare facilities selected and healthcare workers interviewed

Level of facility and staff	Number of facilities and staff selected in each region			
	Ashanti	Central	Northern	Total
Level of facility				
Teaching or Regional Hospital^a^	0	1	1	2
District or Main Hospital	3	2	2	7
Private Hospital or Health Centre	2	2	2	6
Total	5	5	5	15
Healthcare staff				
Regional STI Coordinator^b^	1	1	0	2
Medical Superintendents/Medical Officers	3	5	3	11
Medical Assistants	1	2	1	4
Midwives	6	7	5	18
Laboratory Scientists	4	3	3	10
Pharmacists/Dispensing Technicians	4	1	3	8
Total	19	19	15	53

STI, sexually transmitted infection

^a^The teaching and regional hospitals in the Ashanti region were not selected because: Syphilis test results were not routinely documented in the antenatal care register at the teaching hospital, and antenatal care attendance in the regional hospital was much lower than that of the selected district hospital. ^b^The regional STI coordinator for the Northern Region was not available for interview during our visit to the region

**Table 2:** Thematic framework, personnel interviewed and key findings of investigation of syphilis POC testing policy in Ghana

Themes and sub-themes	Personnel interviewed	Key findings
1. Uptake and successes		
• Syphilis testing for pregnant women	Facility in-charges, midwives and laboratory technologists	Almost all facilities were performing syphilis tests for pregnant women either at the antenatal clinic or laboratory Two facilities could not start syphilis testing due to lack of/expired test kits
• Successes	Facility in-charges, midwives, pharmacists and laboratory technologists	Decentralisation of syphilis testing Free syphilis testing and treatment for pregnant women
2. Staff training and facility level preparedness for rollout of treponemal POCTs		
• Awareness of policy on antenatal syphilis screening	Facility in-charges, medical officers and midwives	All respondents aware of the need to screen all pregnant women for syphilis
• Knowledge of consequences of untreated maternal syphilis	Facility in-charges, medical officers and midwives	Almost all staff knew of an adverse pregnancy outcome due to maternal syphilis Considered knowledge of the consequences of untreated maternal syphilis to be essential in counselling
• Training and retraining in the use of treponemal POCTs	Facility in-charges, midwives, laboratory technologists and STI coordinator	Midwives trained in all facilities Issues with quality of training (compared to HIV training): duration, content, practical demonstration and interpretation of test results Lack of standard operating procedures No re-retraining/refresher training after the initial training
• Antenatal syphilis screening, treatment and referral guidelines	Facility in-charges, midwives, lab technologists and STI coordinator	No guidelines for antenatal syphilis screening Syphilis treatment protocols available in only two facilities No referral guidelines for syphilis screening and treatment Screening and treatment guidelines/protocols for other maternal and newborn interventions displayed at vantage points in facilities No partner notification cards/slips for STIs (including syphilis & HIV)
3. Staff experiences and challenges		
• Maternal syphilis as a component of health education during pregnancy	Midwives	All but one facility discussed maternal syphilis during health education sessions for pregnant women
• Experience of performing HIV and syphilis tests together	Midwives	Easier and time saving for healthcare providers Good counselling required for a clear understanding and interpretation of HIV and syphilis test results Convenient for pregnant women
• Challenges	All	Stockouts of syphilis test kits and benzathine penicillin Inadequate staff/work overload Poor documentation of syphilis test results and treatment Lack of supervision and quality checks

ANC - Antenatal care; POCT – Point of care test; STI - Sexually transmitted infection

### Uptake and successes

With the exception of two health centres in Region 3, all other facilities (13/15) were performing syphilis tests for pregnant women. Although staff in the two facilities were trained to perform the tests, they had never started the testing either because they were not supplied with any test kits or the initial consignment of test kits they got were all expired before any testing could commence:

*“I was trained last year [2009]… I have never done the test [for pregnant women]… They gave us the test kits but when we realized that they were all expired, I sent them back [to the regional medical stores]… We made a request and they said anytime they receive some [test kits] they will supply us.”* (Midwife)

All 13 facilities were using the same syphilis POCT, i.e. Determine syphilis TP (Abbott Japan Co Ltd., Tokyo, Japan) that was supplied by the National AIDS/STI Control Programme and distributed through the regional medical stores. Midwives were performing syphilis tests for pregnant women at the antenatal clinic in all but three facilities (in Region 3), where syphilis testing was done at the laboratory within the facility. Interestingly, while HIV tests for pregnant women were done at the antenatal clinic in the three facilities, insufficient staff cadre was cited as the reason for the inability to perform syphilis tests at the antenatal clinic in those facilities.

The main successes of the rollout programme were reported to be easy integration of syphilis screening into ANC services and its decentralisation to most public health facilities. Antenatal syphilis testing and treatment were offered free of charge to all pregnant women, their babies and partners. Decentralisation of testing to public facilities at all levels of the healthcare delivery system was perceived as a relief to many rural women who could not afford the cost of travel to larger facilities such as the regional hospital when they were referred for testing:

*“The decentralisation has been very helpful. Hitherto patients were travelling far to, say, the regional hospital [more than 120km away] to go and have these tests done and failed to go because of cost [of travel]; so decentralization is the best...”* (Medical superintendent)

Some midwives were advocating that syphilis testing and treatment should be extended to the private maternity homes since they attend to a considerable number of pregnant women especially in rural settings:

*“...instead of referring them to major health centres it should be done at every clinic including the private midwives, since most pregnant women also visit these midwives.”* (Midwife)

### Staff training and facility level preparedness for the rollout of syphilis POCTs

Healthcare staff generally agreed that it was a requirement of the GHS that *“…all pregnant women should be screened for syphilis during antenatal care…”* and stated that such a policy had been in existence for several years. Almost all staff knew of at least an adverse pregnancy outcome associated with untreated maternal syphilis. They contended that knowledge of syphilis was necessary in counselling pregnant women to take up syphilis testing. As one midwife explained:

*“…because I tell them the consequences [of maternal syphilis] and how the baby can be affected just as in HIV… they are willing do it [syphilis test]...”* (Midwife)

Midwives were trained in the use of syphilis POCTs in almost all of the health facilities surveyed. The training was commonly done during HIV testing workshops/review meetings but the form and quality of training varied across districts and regions. In some regions, laboratory personnel were trained at the regional level and the various District Health Management Teams (DHMTs) tasked with organizing training for the midwives at the district or facility level:

*“We did our initial training at the regional level when we were doing the PMTCT and encouraged the DHMTs to arrange for the antenatal service providers to be trained by the lab [laboratory] men and scale up... So this was a package to retrain the lab personnel who will in turn train the midwives.... In one district, the lab men came to the District [Health] Directorate and organized a training session for other staff that had not been trained.”* (STI coordinator)

However, some midwives expressed concern about the quality of the training they received for syphilis testing compared to that of HIV testing especially as they were required to train their colleagues at the various facilities. These midwives did not feel very confident to train other healthcare providers as they considered the training to be short in duration, inadequate in content and lacked practical demonstration of the testing process and interpretation of the test results. Nonetheless, these midwives carried out these training sessions:

*“I was not really trained... it was at a [HIV] review meeting that I was given some tit bits... I won’t call that training! But when I came I taught others; the community health nurse and others now know how to do the tests...”* (Midwife)

The inadequacies of some of the training sessions were corroborated by some laboratory scientists who were involved in the training. Yet, about one year after the initial training, none of the midwives had been retrained and there did not seem to be any plans for such refresher courses. Healthcare providers advocated for adequate training and regular retraining to update their skills. As one laboratory technologist summarised:

*“I think the midwives should be well trained...because anybody at all can decide to do the test... and that can affect the patient. At least once a while, we should retrain those actually doing the tests.”* (Laboratory technologist)

There were no clear guidelines or documents available to midwives on syphilis testing, how to perform the tests, on which subpopulation and when, in any of the facilities visited. However, various guidelines for other maternal and newborn interventions were displayed in most facilities; 11/15, 12/15 and 14/15 of the facilities had HIV testing, IPTp and tetanus toxoid administration guidelines, respectively (Table 3). These guidelines were similar across facilities and were mostly from the GHS. The lack of screening and management guidelines for syphilis was corroborated by one regional STI coordinator:

*“We have [screening and treatment guidelines] for HIV but we do not have specific guidelines for syphilis.”* (STI coordinator)

**Table 3:** Availability of screening/treatment protocols and referral guidelines for antenatal and neonatal interventions

	Number of facilities		
Management Protocol/Guideline	Available, displayed	Available, not displayed	None available
Screening and treatment guidelines/cards (*N* = 15)			
Syphilis screening guidelines^a^	0	0	13
HIV screening guidelines	8	3	4
Malaria IPT administration guidelines	11	1	3
Tetanus toxoid administration guidelines	13	1	1
STI partner notification cards (HIV & syphilis)	0	0	15
Syphilis treatment protocols/cards (*N* = 13)^a^			
Treatment protocol for seropositive mothers	1	1	11
Treatment protocol for babies of seropositive mothers	1	0	12
HIV treatment protocols and referral guidelines			
Treatment protocol for seropositive mothers (*n* = 8)^b^	8	0	0
Treatment protocol for babies of seropositive mothers (*n* = 8)^b^	7	1	0
Referral guidelines for treatment of seropositive mothers (*n* = 7)^c^	0	0	7
Referral guidelines for treatment of babies of seropositive mothers (*n* = 7)^c^	0	0	7

STI, sexually transmitted infection; ^a^2 facilities that had not started screening pregnant women for syphilis were excluded; ^b^7 facilities that were not offering antiretroviral treatment for HIV were excluded; ^c^8 facilities that were offering antiretroviral treatment for HIV were excluded

Only two facilities had treatment protocols for syphilis, one of which also had a protocol for management of neonates (Table 3). Prophylactic treatment was only offered to babies of syphilis seropositive women in the facility with the protocol for neonates. Although most healthcare providers knew of benzathine penicillin as the treatment of choice for syphilis in adults, they could not recall the exact dosage, and were not aware of any national treatment guidelines for syphilis. Consequently, treatment regimens varied from one facility to the other; ranging from a single intramuscular dose of 2.4 M units through three doses given at weekly intervals, to seven doses (single dose daily for 7 days). There were no protocols for managing penicillin allergy, neither were there partner notification cards for STI management including syphilis and HIV (Table 3).

There were no referral guidelines for syphilis screening and treatment and pregnant women were hardly referred from one facility to the other for testing or treatment. Similarly, no referral guidelines were available in facilities that were not offering antiretroviral treatment for HIV. Conversely, IPTp and tetanus toxoid were administered in all facilities and there was no need for referral to other facilities for these interventions.

### Staff experiences and challenges

In all but one facility (where pregnant women were not being screened for syphilis), maternal syphilis was discussed as part of the health education offered during ANC. Other maternal and newborn interventions such as PMTCT of HIV, prevention of malaria in pregnancy and neonatal tetanus were discussed routinely during ANC in all facilities. Health education sessions were conducted in the local dialects, were interactive and preceded any individual counselling, testing or treatment. Midwives noticed that most pregnant women readily opted for counselling and testing for syphilis especially after a good health education session:

*“We give them health education talks in Twi [vernacular] and this helps them to understand. Following this most of the women then come for the test because of the explanation we give them... when we educate the women nicely, they come and do the test.”* (Midwife)

Most training programmes emphasized the need to do HIV and syphilis tests together at the antenatal clinic. The majority of the midwives who did the two tests together acknowledged it was convenient for the client (one instead of two needle pricks and queuing once instead of twice), human resource sparing and time saving for healthcare providers (one person doing the two tests at the same time thus using less time than would be required by two different individuals to do the two tests separately) and quite easy (tests procedure and interpretation very much similar), and advocated the practice should be encouraged. One midwife shared her experience of doing the two tests simultaneously:

*“I have not had any problems because we counsel them well. ...what I do is that I prick the women once and put a drop of blood each on the syphilis and HIV test strips add their buffers and wait for the results... They understand because I explain everything to them before I do the test... Sometimes they ask a lot of questions... No they will not get confused [with HIV and syphilis results]... it is easy [to do the two tests together] and we should do them at the same time.”* (Midwife)

Structured observations made during our visits showed that pre-test counselling for HIV or syphilis were generally satisfactory; staff were friendly, gave explanations as necessary and allowed clients to seek clarifications. However, management of the few syphilis- or HIV-seropositive women (two syphilis- and three HIV-seropositive women) during our visits could generally be described only as “fair” as most of the issues related to providing comprehensive treatment such as eliciting symptoms and risk factors, undertaking partner notification, and prophylactic treatment for the baby at birth were not discussed in sufficient detail.

Within three months of starting syphilis screening, most facilities (8/13) had to suspend testing for 1–3 months due to “expired test kits” and to a lesser extent stockouts (of unexpired test kits). Some facilities had barely started using the test kits at antenatal clinic when they had to suspend testing. This was recognised at the facility and regional levels, and largely attributed to the procurement system:

*“We started testing [at the antenatal clinic] towards the end of June [2010] and stopped early July [2010]... The test kits that we had got expired, so we could not continue... I tried [contacted] the [medical] stores but I could not get any.”* (Midwife)

*“…The first one [challenge] has to do with the supply of [syphilis] test kits, which as I have already mentioned, is erratic. Another problem is that the time between receiving these commodities and the expiry dates is so short. Sometimes we get [syphilis] test kits that are expiring within six months… I think it’s the interval of ordering. Sometimes they order them and by the time they reach here and are supplied they are almost nearing expiring dates.… I think the whole problem has to do with the procurement system.”* (STI coordinator)

From our stock audits, almost half (6/13) of facilities which had started antenatal syphilis screening did not have any syphilis test kits while HIV test kits were available in all the 14 facilities that were screening pregnant women for HIV (Table 4). In some facilities, stockouts of syphilis test kits were quite frequent but pregnant women were not referred to other facilities for testing, and were only screened when the test kits became available. In one region, while healthcare providers blamed stockouts of test kits on inadequate supplies from the regional level, staff at the regional directorate blamed it on the lack of returns from the districts and healthcare facilities (facilities are required to submit periodic returns/reports on all supplies provided to them), and less commonly insufficient supplies from the Central level (national headquarters):

*“We [at the Regional Health Directorate] have not received any feedback; I have a problem here because they should have sent us returns indicating how many test kits have been used, how many got expired. They should have made contact with their facility heads who will in turn contact the District Director [of Health Services] and then the region will be informed. I don’t think we have to wait for Accra [National AIDS/STI Control Programme] to supply us... none of them [facilities] gave us information on shortages or expired kits, and once expired, the expired kits need to be returned to the regional medical stores so that feedback can be given to the national level.”* (STI coordinator)

**Table 4:** Stock levels of test kits, lancets, drugs and needles and syringes for HIV and syphilis testing and treatment

Consumables	Stock levels		
	Nil	< 1 month	≥1 month
Test kits and lancets			
Syphilis test kits (*n* = 13)^a^	6	0	7
Syphilis test kits-chase buffer (*n* = 13)^a^	6	1	6
HIV test kits/diluent (*n* = 14)^b^	0	4	10
Lancets (*n* = 15)	1	0	14
Drugs and needles and syringes			
Injection Benzathine penicillin (*n* = 13)^a^	8	1	6
Tablets Erythromycin (*n* = 13)^a^	7	0	8
Syrup Nevirapine (*n* = 8)^c^	0	2	6
Tablets Sulphadoxine pyrimethamine (*n* = 15)	2	1	12
Needles and syringes (*n* = 15)	1	0	14

^a^2 facilities which had not started syphilis screening were excluded; ^b^1 facility which had not started antenatal HIV screening was excluded; ^c^7 facilities that were not offering antiretroviral therapy (nevirapine) to babies of HIV positive mothers and referred all babies to other facilities for treatment were excluded

On the other hand, stockouts of benzathine penicillin were uncommon. Only one hospital reported stockouts, but was not aware that requisition had to be made on a special form, as the drug was regarded as a “programme drug” (i.e. meant for only the syphilis programme) at the regional level and healthcare facilities ought to have been informed accordingly*.* Unfortunately, pregnant women with a positive syphilis serology had to purchase benzathine penicillin on their own from private shops within the period of the stockout.

Our audit revealed that almost half (6/13) of the facilities had not been supplied with benzathine penicillin since routine syphilis testing started. Some facilities (5/13) had also not been given erythromycin as alternative treatment in cases of penicillin allergy. On the other hand, nevirapine was available in all facilities that offered prophylactic treatment to babies of HIV seropositive mothers. Sulphadoxine pyrimethamine for IPTp was lacking in only two facilities and stockouts of needles and syringes were less common (Table 4).

Staff numbers were considered to be insufficient in most facilities, and midwives complained of increased workload especially when they had to complete additional registers or do extra “paper work”:

*“...a lot of writing; filling the insurance [National Health Insurance forms], antenatal book, registers, prescription etc and that consume a lot of time. So we need more staff...”* (Midwife)

Nevertheless, most midwives considered syphilis testing as an integral part of their duty, and were willing to do the tests. In a few “one healthcare provider” stations, clients had to spend long waiting times for their turn to do the test or the test could not be offered at all times.

Although most midwives were willing to do the tests, both the facility managers and midwives agreed that it was necessary to encourage and motivate the midwives for the “extra work” being done to ensure that the spirit and enthusiasm with which they started the testing did not die down. Money was commonly suggested as the form of motivation; other suggestions were commendation for good work, training workshops, gifts, print or electronic material on syphilis, and uniforms or polo shirts. Generally, it was agreed that motivational packages could be funded from internally generated funds and given monthly, quarterly, half-yearly or yearly:

*“Motivation must not always be financial; any package that will show appreciation for the good work being done like citation and further training will motivate them to improve upon their performance... These can even be given at the end of the year.”* (Medical assistant)

All four prenatal interventions were routinely recorded in the maternal record booklets in all facilities, but recording in the antenatal care registers was very variable. There was no column in the antenatal care register for recording syphilis treatment. In some instances, midwives complained of *“too much”* documentation, partly due to the existence of multiple registers for different programmes for example different registers for recording HIV results, tetanus toxoid and IPTp.

No quality control programme or monitoring and supervisory visits were instituted to monitor the progress of the programme. These were all proposed by healthcare providers during our study. Significantly, midwives felt the need for their testing to be supervised and laboratory personnel were also happy to provide internal checks:

*“...Once in a month there should be some quality control checks; we have positive and negative samples, we can take them to the clinic for the midwives to use their kits on... Occasionally we can also pass-by and see what the midwives are doing.”* (Laboratory technologist)

Healthcare providers were quite enthusiastic about new dual POCT kits, i.e. combining HIV and syphilis tests, or treponemal and non-treponemal tests in the same testing cassette. The former will enhance the integration of screening for both infections into ANC, while the latter, by distinguishing active syphilis infections from past/treated syphilis infections or individuals without syphilis infection will streamline treatment. The providers presumed that these newer kits would be easy to use and if offered free of charge, would be particularly helpful at antenatal clinics. Apart from saving staff and clients’ time, these tests would also prevent the need for multiple needle pricks or blood samples, especially when midwives forget to take blood specimen for one test or the other. As one midwife put it:

*“It will be helpful. Sometimes you forget you are going to do the syphilis test after you have taken the blood drop for HIV. So after the HIV test you have to prick the woman again. So if the two tests are on the same test kit you will remember to do the two [tests] at the same time”* (Midwife)

## Discussion

This study focused on the successes of rolling out syphilis POCTs in Ghana and identified challenges to the implementation process. Although, routine syphilis screening was integrated into ANC services and decentralised, key challenges such as inadequate training and lack of updates/refresher courses, lack of testing guidelines and treatment protocols, frequent stockouts/expired test kits due in part to lack of clear communication between central and periphery units, increased workload and poor documentation of test results and treatments in the ANC register needed to be addressed to improve and sustain the use of this new technology in Ghana.

Our study highlighted the benefits of decentralisation of free antenatal syphilis screening with POCTs in this low resource setting. Most public health facilities had started free antenatal syphilis screening, thereby making testing more accessible especially to the rural folks. Since ANC coverage is high (> 90%) [8], effective rollout of syphilis POCTs has the potential of reaching almost all pregnant women. It is imperative that use of syphilis POCTs must be extended to private health facilities if the goal of achieving universal access to antenatal syphilis screening and treatment is to be attained. In the meantime, a policy of “mop-up” screening could be instituted at delivery where all pregnant women who were not screened during ANC will be tested at delivery and treated as necessary; partner notification and treatment could be offered during postnatal visits. Whilst syphilis testing at delivery cannot prevent vertical transmission in the index pregnancy, it provides an opportunity to offer prophylactic treatment to such babies (unless there are signs of congenital syphilis in which case full treatment will be required) and may prevent vertical transmission in subsequent pregnancies. However, this does not preclude prenatal testing during future pregnancies.

Knowledge of the policy on an intervention is a prerequisite for the acceptance and successful implementation of the policy at the facility level [13]. Healthcare providers were aware of and accepted the policy to screen all pregnant women for syphilis during ANC. Counselling skills were enhanced by a good understanding of the consequences of maternal syphilis in this study. These findings suggest that knowledge and an understanding of a policy/programme can greatly enhance its implementation. In South Africa, Sprague et al. [14] observed that health workers’ poor knowledge on PMTCT resulted in inadequate counselling which adversely affected the PMTCT programme.

Generally, introduction of any new technology such as rapid syphilis POCTs should only start after sufficient stakeholder consultation, the development of clear testing and treatment guidelines, comprehensive training, a reliable test and drug supply, and establishment of clear communication channels to report issues in implementation and ensure no stockouts. Our study revealed that most training sessions were inadequate in content, lacked practical demonstrations and refresher training workshops had not been organised. Training sessions should include laboratory practical on the test procedure and interpretation of results, how to train and assess competence of healthcare staff, supply chain procedures, stock management, record keeping and quality control [3, 15]. Integrating such training sessions into existing HIV PMTCT training workshops would save cost and healthcare providers time [15]. It must however be emphasized that similarity in the operating principles of HIV and syphilis POCTs does not preclude adequate training prior to rollout. As much as possible, implementation projects should be piloted to identify and address key challenges before scale-up [3, 15].

On the other hand, inadequate training, insufficient guidance and supervision and lack of a quality assurance system would adversely affect the quality of testing [3, 15]. Both ANC and laboratory staff recognised the need to complement one another’s effort to improve testing but no quality assurance and monitoring systems were in place to monitor the performance of the programme.

Refresher training is an essential component of any programme implementation and has been shown to impact positively on scale-up of antenatal syphilis screening [3, 16]. Given the insufficiency of the initial training workshops, refresher courses should have been integrated into regular PMTCT workshops to update and improve staff knowledge and skill in the use of syphilis POCTs.

Guidelines for testing and treatment protocols are important aspects of syphilis POCTs rollout. As much as possible these should be standardized with those for PMTCT to simplify procedures for staff, ensure consistency and ease of implementation and provide quality care to clients [15]. Luckily, national testing guidelines and treatment protocols for HIV were available in most facilities; these could have been adapted for syphilis testing and treatment. Notwithstanding the similarity in testing procedures, treatment protocols for HIV and syphilis are quite different emphasizing the need to provide separate clear guidelines for syphilis treatment or remind healthcare staff to refer to the Ministry of Health’s Standard Treatment Guidelines [17] during training sessions. For example, the Standard Treatment Guidelines recommends a single dose of 2.4 M units (MU) of benzathine penicillin for all stages of syphilis [17], and not the multiple and variable doses that were being given in some facilities. Lack of screening and treatment guidelines adversely affected the implementation of antenatal syphilis screening in Kenya and Bolivia [18].

Interruptions in the supply of syphilis POCTs and penicillin resulted in missed opportunities to prevent congenital syphilis. Our study provides insights into how lack of clear communication channels and poor monitoring and supervision adversely affected implementation of the programme. Shortages in syphilis test kits were attributed to expired tests kits and failure to replenish stocks. Interestingly, healthcare providers and programme coordinators blamed each other for stockouts. This demonstrates a lack of clear communication channels and inadequate monitoring and supervision of the programme; factors which were partly responsible for poor implementation of the policy previously [5]. Discussing supply chain procedures and stock management during training sessions and linking the procurement of syphilis-related stocks to those of PMTCT could have resulted in a more consistent supply of syphilis test kits and penicillin [3, 19], as stockouts of HIV test kits were reported to be less frequent.

In an earlier study in Mozambique, Gloyd et al. [16] reported that the existence of multiple registers coupled with workload impacted negatively on the recording of syphilis test results and other interventions in the ANC register. Unfortunately, syphilis treatment uptake could not be evaluated in this study due to the absence of a column for syphilis treatment in the ANC register. However, contemporary studies from Uganda and Zambia have demonstrated that ANC registers can be modified to record antenatal syphilis screening and treatment by adding extra columns [15]. Moreover, keeping a single “integrated register” makes recording easier for providers, saves time and makes it simpler to monitor integrated programmes concurrently [15].

In most low-income countries, inadequate numbers of staff and workload can be key challenges during the implementation of priority interventions [19, 20]. There is often the need to provide fiscal and/or non-monetary incentives [20, 21]. Following the rollout of syphilis POCTs, healthcare staff felt the need to be motivated and suggested both financial and non-financial incentives such as refresher workshops/further training, emphasising the need to consider motivation beyond money. Refresher training has the additional benefit of improving staff knowledge and skill in the particular intervention. Therefore, the appropriate mix of incentives should be identified to maximize staff satisfaction.

It is worth noting that healthcare providers were positive about using new dual syphilis test kits, indicating their willingness to embrace the newer technology. HIV/syphilis dual test kits will strengthen integration of both tests into ANC and ensure that pregnant women are screened for both infections at the same time. The new dual treponemal/non-treponemal POCTs [22] will be very useful at antenatal clinics in the country especially in areas endemic for yaws. These dual test kits can correctly identify infection status (active, past/treated or absent) within a few minutes and avoid overtreatment of past/treated infections [22].

The study had a number of limitations. First, the selection of three regions (out of 10) may not have adequately reflected on the countrywide experience of syphilis POCTs rollout. Nonetheless, the three regions were geographically representative and reflect different levels of maternal syphilis seroprevalence in the country, which in turn may have impacted on levels of interest and proficiency in syphilis testing. Second, one of the three regional STI coordinators was not available for interview; coincidentally, this was in the region with the lowest syphilis prevalence. Although his experiences would have been helpful, the other two coordinators gave a fair idea of the implementation and collaboration between the Regional, District and facility levels. Third, it is possible that the presence of the research team especially the main investigator, could have modified staff attitude and practice as a result of being observed or resulted in self-censoring answers (the so-called ‘Hawthorne effect’) [23]. Although this study was conducted a while ago, the general lessons are useful for the introduction of any new technology within the health systems of countries like Ghana. Meanwhile, some remedial action has been taken; the two facilities that hitherto were not offering antenatal syphilis screening have started using syphilis POCTs. Also, a new ANC register with a column for syphilis treatment is in the pipeline. However, major challenges relating to stockouts of and expired test kits, availability of benzathine penicillin, testing and treatment guidelines, clear communication channels, retraining and monitoring and supervision largely remain unresolved.

## Conclusion

Although syphilis POCTs were integrated into ANC services, it appears the rollout focused on the purchase and distribution of test kits and drugs, and preliminary training of healthcare providers; which were largely rushed and perhaps inadequate to meet the challenges of a sustainable programme. Key challenges particularly around supply chain management procedures, establishment of clear channels of communication, quality assurance and supervision needed to be addressed to improve and sustain the programme. New technologies can make an impact when existing challenges are addressed first. A review of the rollout as well as addressing the major challenges identified above would greatly improve the programme.

## Abbreviations

ANC: antenatal care
DHMT: District Health Management Team
GHS: Ghana Health Service
IPT_p_: intermittent preventive treatment of malaria in pregnancy
MU: mega units
POCTs: point of care tests
RPR: rapid plasma reagin
STIs: sexually transmitted infections
VDRL: Venereal Disease Research Laboratory
